# A novel cyclic biased agonist of the apelin receptor, MM07, is disease modifying in the rat monocrotaline model of pulmonary arterial hypertension

**DOI:** 10.1111/bph.14603

**Published:** 2019-04-01

**Authors:** Peiran Yang, Cai Read, Rhoda E. Kuc, Duuamene Nyimanu, Thomas L. Williams, Alexi Crosby, Guido Buonincontri, Mark Southwood, Stephen J. Sawiak, Robert C. Glen, Nicholas W. Morrell, Anthony P. Davenport, Janet J. Maguire

**Affiliations:** ^1^ Experimental Medicine and Immunotherapeutics University of Cambridge Cambridge UK; ^2^ Department of Medicine University of Cambridge Cambridge UK; ^3^ Wolfson Brain Imaging Centre, Department of Clinical Neuroscience University of Cambridge Cambridge UK; ^4^ Department of Pathology Papworth Hospital NHS Foundation Trust Cambridge UK; ^5^ The Centre for Molecular Informatics, Department of Chemistry, University of Cambridge, Cambridge UK and Computational and Systems Medicine, Department of Surgery and Cancer, Faculty of Medicine, Imperial College London UK

## Abstract

**Background and Purpose:**

Apelin is an endogenous vasodilatory and inotropic peptide that is down‐regulated in human pulmonary arterial hypertension, although the density of the apelin receptor is not significantly attenuated. We hypothesised that a G protein‐biased apelin analogue MM07, which is more stable than the endogenous apelin peptide, may be beneficial in this condition with the advantage of reduced β‐arrestin‐mediated receptor internalisation with chronic use.

**Experimental Approach:**

Male Sprague–Dawley rats received either monocrotaline to induce pulmonary arterial hypertension or saline and then daily i.p. injections of either MM07 or saline for 21 days. The extent of disease was assessed by right ventricular catheterisation, cardiac MRI, and histological analysis of the pulmonary vasculature. The effect of MM07 on signalling, proliferation, and apoptosis of human pulmonary artery endothelial cells was investigated.

**Key Results:**

MM07 significantly reduced the elevation of right ventricular systolic pressure and hypertrophy induced by monocrotaline. Monocrotaline‐induced changes in cardiac structure and function, including right ventricular end‐systolic and end‐diastolic volumes, ejection fraction, and left ventricular end‐diastolic volume, were attenuated by MM07. MM07 also significantly reduced monocrotaline‐induced muscularisation of small pulmonary blood vessels. MM07 stimulated endothelial NOS phosphorylation and expression, promoted proliferation, and attenuated apoptosis of human pulmonary arterial endothelial cells in vitro.

**Conclusion and Implications:**

Our findings suggest that chronic treatment with MM07 is beneficial in this animal model of pulmonary arterial hypertension by addressing disease aetiology. These data support the development of G protein‐biased apelin receptor agonists with improved pharmacokinetic profiles for use in human disease.

**Abbreviations**
CHX: cycloheximide
EBM‐2: endothelial basal medium 2
EGM‐2: endothelial growth medium 2
LV: left ventricle
MCT: monocrotaline
PI: propidium iodide
PAECs: pulmonary artery endothelial cells
PAH: pulmonary arterial hypertension
PMVECs: pulmonary microvascular endothelial cells
rhVEGF: recombinant human VEGF
RV: right ventricle
RVSP: right ventricular systolic pressure

**What is already known**

- Apelin is down‐regulated in pulmonary arterial hypertension.

**What this study adds**

- A G protein‐biased apelin peptide agonist is disease‐modifying in a model of pulmonary arterial hypertension.

**What is the clinical significance**

- Supports the development of G‐protein‐biased apelin agonists for treatment of pulmonary arterial hypertension.

## 1 INTRODUCTION

The G protein‐coupled apelin receptor (Alexander et al., 2017) is emerging as a novel therapeutic target for pulmonary arterial hypertension (PAH; Yang, Maguire, & Davenport, 2015) with evidence of the beneficial effect of enhancing apelin receptor signalling in PAH, supported by a small number of studies from PAH patients and animal models. Expression of its endogenous ligand apelin is reduced in the circulation of patients with Group 1 pulmonary hypertension, including idiopathic PAH, PAH associated with drugs or toxins, and autoimmune disease (Chandra et al., 2011; Goetze et al., 2006). In the pulmonary vasculature, pulmonary artery endothelial cells (PAECs; Kim et al., 2013) and pulmonary microvascular endothelial cells (PMVECs; Alastalo et al., 2011) from PAH patients express lower levels of apelin compared to cells from control donors. Mice lacking apelin develop more severe pulmonary hypertension under hypoxia (Chandra et al., 2011). Nevertheless, the apelin receptor is still present in PAH tissue (Andersen, Markvardsen, Hilberg, & Simonsen, 2009; Kim et al., 2013, Yang, Read, et al., 2017) and would therefore be available as a target for exogenous agonists to replace the missing apelin. This hypothesis is substantiated by the emerging evidence that apelin may exert a range of protective effects in PAH. Apelin knockout mice develop higher right ventricular systolic pressure (RVSP), increased muscularisation of the alveolar wall arteries, and more loss of pulmonary microvasculature in response to hypoxia, compared to wild‐type controls (Chandra et al., 2011). Administration of the apelin peptide (Alastalo et al., 2011; Falcão‐Pires et al., 2009) or other agents stimulating apelin expression (Bertero et al., 2014; Nickel et al., 2015; Spiekerkoetter et al., 2013) or downstream mediators of the apelin pathway (Kim et al., 2013) has demonstrated benefits in animal models of PAH.

Studies to date have administered the native isoforms of the apelin peptides to animal models of PAH. For example, [Pyr^1^]apelin‐13, the most predominant isoform in the human cardiovascular system (Maguire, Kleinz, Pitkin, & Davenport, 2009), was tested in monocrotaline (MCT)‐induced PAH in rat (Falcão‐Pires et al., 2009). As an endogenous ligand, [Pyr^1^]apelin‐13 activates both G protein and β‐arrestin‐mediated signalling of the apelin receptor. Activation of the β‐arrestin pathway results in receptor desensitisation and internalisation, leading to loss of efficacy with chronic use (Evans et al., 2001). Importantly, β‐arrestin‐mediated signalling of the apelin receptor may cause stretch‐induced myocardial hypertrophy and heart failure, whereas apelin‐induced G_αi_ signalling is protective (Scimia et al., 2012). These reports suggest that minimising β‐arrestin recruitment while maintaining G protein signalling would be desirable for the apelin receptor. This can be achieved by using a G protein‐biased apelin receptor agonist.

**Figure 1:**
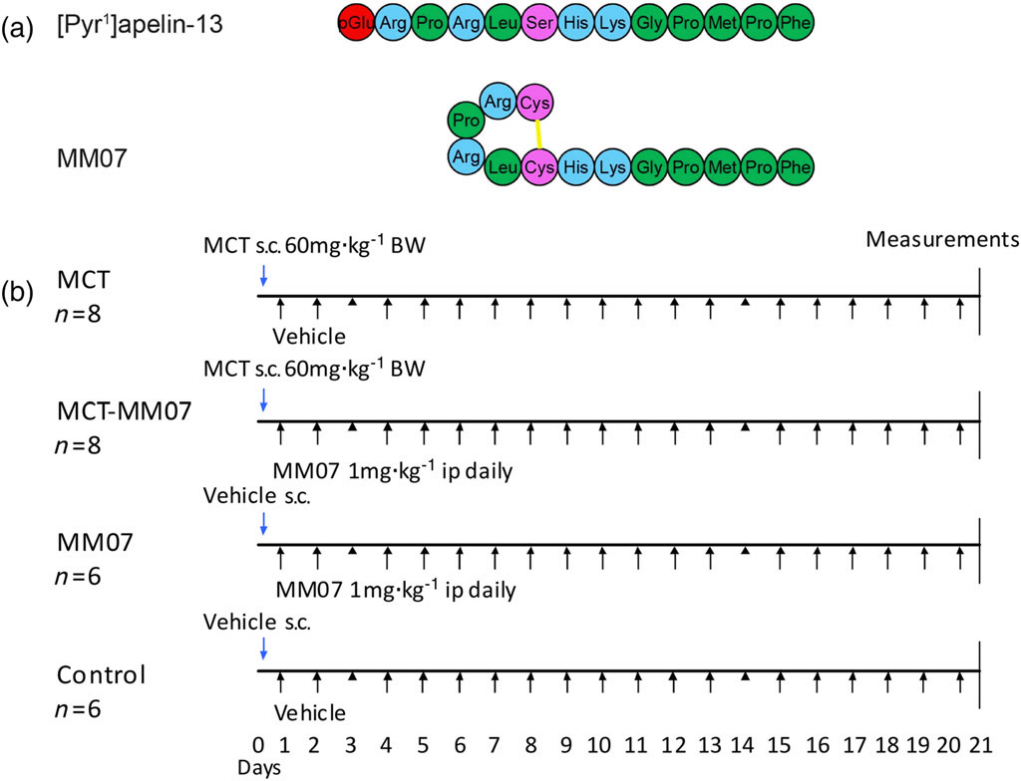
(a) Amino acid sequences of MM07 aligned with the sequence of the endogenous agonist [Pyr^1^]apelin‐13. The disulfide bridge is shown as yellow lines, hydrophobic amino acids shown in green, uncharged polar amino acids in pink, and basic amino acids in blue with pyroglutamate in red. (b) Experimental design of the MCT‐MM07 study. Rats were given monocrotaline (MCT; 60 mg·kg^−1^) or vehicle on Day 0 and daily injections of MM07 (1 mg·kg^−1^) or vehicle from Day 1 to Day 20. Cardiac MRI was conducted on Day 20 and closed‐chest catheterisation was performed on Day 21. The animals were then killed for tissue collection. BW, body weight

We have examined signalling bias of our apelin peptide analogues designed using a combination of computational modelling and pharmacological assays and identified MM07 as a candidate for the first synthetic biased apelin receptor agonist (Brame et al., 2015). The N terminal cyclised structure of MM07 (Figure 1a) was designed to constrain the peptide to specific conformations and protect against enzymic degradation by non‐specific aminopeptidases. Molecular dynamics simulations showed that MM07 was expected to mimic the solution conformation of apelin‐13 and promote a β‐turn conformation at the RPRL motif, suggested to be important for initial recognition and binding at the apelin receptor (Brame et al., 2015; Macaluso & Glen, 2010). MM07 was two orders of magnitude less potent than [Pyr^1^]apelin‐13 in the cell‐based β‐arrestin and internalisation assays but was equipotent to [Pyr^1^]apelin‐13 in a G protein‐dependent saphenous vein constriction bioassay (Brame et al., 2015). Based on these results, MM07 was found to be a G protein‐biased apelin receptor agonist. In anaesthetised rats, MM07 infusion caused a greater increase in cardiac output than [Pyr^1^]apelin‐13 and MM07 had an in vivo plasma half‐life sevenfold longer than that of [Pyr^1^]apelin‐13, possibly owing to the cyclisation and also to the reduced internalisation of MM07 bound apelin receptor (Brame et al., 2015). In first‐in‐human experiments, MM07 was more efficacious than [Pyr^1^]apelin‐13 at increasing forearm blood flow and, consistent with G protein versus β‐arrestin bias, did not exhibit loss of efficacy with repeated administration (Brame et al., 2015). Additionally, both [Pyr^1^]apelin‐13 and MM07 reversed an established noradrenaline‐induced constriction in human hand veins (Brame et al., 2015). The aim of the present, proof‐of‐concept study was to test MM07 in the well‐established MCT‐induced model of PAH. We hypothesised that MM07 would effectively attenuate MCT‐induced PAH development.

## 2 METHODS

### 2.1 Animals

All animal care and rodent experiments complied with the Home Office (UK) guidelines under the Animals (Scientific Procedures) Act 1986 Amendment Regulations (SI 2012/3039) and were approved by the local ethics committee (University of Cambridge Animal Welfare and Ethical Review Body). Animal studies are reported in compliance with the ARRIVE guidelines (Kilkenny, Browne, Cuthill, Emerson, & Altman, 2010) and with the recommendations made by the *British Journal of Pharmacology*. All animal experiments were performed and analysed under blinded conditions.

As an animal model of PAH, rat is the preferred species, relative to the mouse, because the anatomical and functional features of rat models are closer to those of the human disease. The MCT rat model exhibits pulmonary vascular remodelling and raised RVSP in addition to dysfunction of airways and alveoli (Colvin & Yeager, 2014). Although MCT rats do not develop plexiform‐like lesions, the changes reported in proximal bronchovascular structures are very similar to aspects of the human condition. Moreover, the MCT model remains the only animal model of PAH that has successfully translated a therapeutic agent from the laboratory to the clinic. This was bosentan, the first endothelin receptor antagonist.

### 2.2 Experimental rat model of PAH

Attenuation of PAH pathogenesis by the apelin receptor agonist MM07 was tested in MCT‐exposed rats using an experimental design similar to that reported by Falcão‐Pires et al., 2009 (Figure 1b). Male Sprague–Dawley rats (186 ± 2 g, Charles River Laboratories [RRID:RGD_734476]) were used at a relatively young age of approximately 7 weeks, which is required for the successful induction of PAH with MCT. The power calculation determining the number of animals used is as follows: *n* > 2{[(*Zα* + *Zβ*)*s*]/*δ*}2, where *n* is the sample size, *Zα* = 1.96, *Zβ* = 1.28 for a study with 90% power and *P* < 0.05 significance level, *s* is SD, and *δ* is the minimum difference. *δ* is set at 20% from a sample with an SD of 10%, based on our previous data. The calculation gives *n* > 5.2. The initial size of the MCT‐exposed groups was larger than the vehicle control group to account for possible attrition over the treatment period.

Rats were randomly allocated initially into two groups: the MCT groups (*n* = 16) were given MCT (60 mg·kg^−1^ body weight; Crotaline cat. no. C2401) with the saline vehicle control group (*n* = 12) receiving an equal volume of vehicle (0.9% saline) by s.c. injection on Day 0. From Day 1 to Day 20, the MCT groups were further divided such that eight rats were randomised to receive daily i.p. injections of MM07 (1 mg·kg^−1^ body weight) and eight to receive an equal volume of vehicle (0.9% saline) daily. Similarly, the vehicle control animals were randomised either to receive daily injections of MM07 (1 mg·kg^−1^ body weight i.p., *n* = 6) or vehicle (0.9% saline i.p., *n* = 6) resulting in four treatment groups in total (Figure 1b). The dose of MM07 was selected based on the previously published study (Falcão‐Pires et al 2009) that found daily injections of 200 μg·kg^−1^ of [Pyr^1^]apelin‐13 attenuated MCT‐induced PAH and the lower affinity and potency of MM07.

The rats were accommodated under standard housing and husbandry conditions, under room temperature (20‐24°C) and normoxia, in individually ventilated cages of four rats each with wood chip bedding, under 12‐hr light/12‐hr dark cycle with ad libitum access to clean water and normal chow. The rats were monitored daily for signs of sickness and distress. No humane endpoint was met during the study.

### 2.3 Cardiac MRI

MRI was carried out as previously described (Buonincontri et al., 2013) to assess cardiac performance on Day 20 in a randomly selected subset of animals. The rats were anaesthetised with inhaled isoflurane for induction (3% in 1.5 L·min^−1^ oxygen) and maintenance (2–2.5% in 1.5 L·min^−1^ oxygen). A pressure sensor for respiration rate was used to monitor depth of anaesthesia, with respiration rate maintained at 45–55 breaths·min^−1^. Body temperature was monitored using a rectal thermometer and maintained at 37°C using a flowing‐water heating blanket. Prospective gating of the MRI sequences was achieved with electrocardiography monitoring using paediatric electrocardiography electrodes (3 M Europe, Diegem, Belgium) on left and right forepaws. MRI was performed at 4.7 T with a Bruker BioSpec 47/40 system (Bruker Inc., Ettlingen, Germany). A birdcage coil of 12 cm was used for signal excitation, and animals were positioned prone over a 2‐cm surface coil for signal reception. After initial localisation images, four‐chamber and two‐chamber views were obtained. Using these scans as a reference, short‐axis slices were acquired (FISP, TR/TE 6 ms/2.1 ms, 20–30 frames, 5‐cm FOV, 256 × 256 matrix, 2‐mm slice thickness, bandwidth 78 kHz, flip angle 20°, NEX 3), perpendicularly to both the long‐axis views. Full ventricular coverage in the short axis was achieved with no slice gap with 9–10 slices. Delineation of the LV and RV and calculation of ventricular volumes and ejection fraction were performed as described using Segment v1.9 (Buonincontri et al., 2013; Heiberg et al., 2010). Owing to limitations in access to MRI facilities, only six each of the MCT/saline and MCT/MM07 groups and four each of the saline and MM07 control animals were imaged, with image quality of one of the MCT animals suboptimal to include in subsequent analysis. Group data for the MCT/saline and MCT/MM07 groups were compared using two‐tailed Student's *t* test. The rats were allowed to recover from anaesthesia after MRI, and the i.p. injection for Day 20 was given.

### 2.4 Haemodynamic assessment by catheterisation

On Day 21, all rats were weighed and catheterised for RV haemodynamic measurements as previously described (Crosby et al., 2010; Long et al., 2015). Briefly, rats were anaesthetised with isoflurane (3% for induction, 2–2.5% for maintenance, and 1.5 L·min^−1^ oxygen). Body temperature was maintained at 37°C. A pressure volume catheter (SPR‐869, Millar, Houston, TX, USA) connected to the data PowerLab 16/35 system (RRID:SCR_001620) with LabChart 5 and calibrated using the MPVS Ultra PV Unit (RRID:SCR_016179) was inserted into the RV via the right external jugular vein to measure RVSP as a surrogate for pulmonary arterial pressure. The position of the catheter was determined by the BP and shape of the pressure volume loops. The animal was allowed to stabilise for measurements. Data analysis was performed using LabChart 8 as previously described (Yang, Read, et al., 2017). Group data were compared using one‐way ANOVA with Tukey's post test.

### 2.5 Analysis of cardiac and pulmonary vascular morphometry

At the end of the catheterization procedure, animals were killed by exsanguination; the left lung was infused with 0.8% agarose to inflate, removed, fixed in 10% formalin (cat. no. BAF‐5000‐08A, CellPath, Powys, UK), paraffin embedded, and stained for smooth muscle α‐actin and elastic van Gieson stain, as described previously (Crosby et al., 2010; Long et al., 2015). The heart was then excised, the RV was dissected from the LV + septum, and the weight ratio of these (RV compared to LV + septum), also known as the Fulton index, was determined as a measure of RV hypertrophy. In the left lung, small (diameter 25–75 μm, 100 per section) pulmonary blood vessels associated with alveolar ducts were scored as completely muscular, partly muscular, or non‐muscular. Statistical significance was assessed by comparing the percentage of fully muscularised vessels between groups. The wall thickness of small pulmonary arterioles (20 per section) close to terminal bronchioles was determined by measuring the average wall thickness as a percentage of average lumen diameter of the vessel using ImageJ (Fiji, RRID:SCD_002285) as previously described (Yang, Read, et al., 2017). Quantification was only performed in samples where the lungs were fully inflated, and fixation was optimal to preserve structure and to allow accurate visualisation of vascular remodelling. Therefore, data for analysis were obtained from a subset of animals (*n* = 6 each for MCT/saline and MCT/MM07 groups and *n* = 4 each for the saline and MM07 controls). Therefore, MCT/saline and MCT/MM07 data only were compared using Student's two‐tailed *t* test.

### 2.6 Regulation of pulmonary arterial endothelial cell signalling

Apelin signalling is known to regulate endothelial NOS (eNOS), a critical enzyme that is thought to be protective in PAH and AMP‐activated protein kinase (AMPK), a regulator of energy metabolism and angiogenesis (Chandra et al., 2011; Yang et al., 2014). We have tested whether MM07 could modulate these two targets. Human PAECs (cat. no. CC‐2530, Lonza, Basel, Switzerland; passages 4–5) were seeded in 96‐well plates (Corning Life Sciences, New York, USA) in endothelial growth medium 2 (EGM‐2, Lonza) with 10% FBS (Gibco™ Thermoscientific, Paisley, UK), allowed to attach overnight, and starved for 6 hr in endothelial basal medium 2 (EBM‐2; Lonza) with 0.5% FBS to establish baseline. The cells were treated for 15 min at 37°C with PBS, [Pyr^1^]apelin‐13 (100 nM) or MM07 (1 μM). Phosphorylated eNOS and AMPKa were detected using a previously described high throughput in‐cell Western assay (Nikolic et al., 2018). The cells were fixed and permeabilised in cold 1% paraformaldehyde with 0.5% Triton‐X for 20 min, washed, and blocked in PBS with 2% BSA (Fisher Scientific, Loughborough, UK) for 2 hr at room temperature. A primary antibody against Serine‐1177 phosphorylated eNOS (RRID:AB_329837, Cell Signaling Technology, 1:1000 in PBS with 2% BSA, *n* = 11 independent experiments) or Thr‐172 phosphorylated AMPKa (RRID:AB_331250, Cell Signaling Technology, 1:200 in PBS with 2% BSA, *n* = 9 independent experiments) was applied for overnight incubation at 4°C. Following PBS washes, a secondary anti‐rabbit IgG HRP‐conjugated antibody (RRID:AB_2099233, Cell Signaling Technology, 1:1000 in PBS with 2% BSA) was added for 1‐hr incubation at room temperature. Following washes, enhanced chemiluminescence (Surmodics) measurement was performed (SpectraMax L, Molecular Devices, San Jose, CA, USA).

In a separate set of experiments, human PAECs (Lonza, passages 4–5, *n* = 9 independent experiments) were seeded in 6‐well plates (Corning) with 10% FBS (Gibco Thermoscientific), allowed to attach overnight, and starved for 6 hr in EBM‐2 (Lonza) with 0.5% FBS. The cells were then treated for 24 hr at 37°C with PBS, [Pyr^1^]apelin‐13 (1 μM), or MM07 (10 μM). RNA extraction was performed using a Trizol®‐based protocol according to the manufacturer's instructions (PureLink® RNA mini kit, cat. no. 1218325, Invitrogen, Carlsbad, CA, USA). Reverse transcription was performed with 500‐ng RNA according to the manufacturer's instructions (SuperScript™ IV VILO™ master mix, cat. no. 11766050, Invitrogen), and quantitative real‐time PCR (qPCR) was performed for NOS3 (gene encoding eNOS) using 18S as internal control (AzuraView™ GreenFast qPCR Blue mix, cat. no. AZ‐2420, Azura Genomics, Raynham, MA, USA; Mastercycler Realplex2, Eppendorf). The primer sequences are as follows: 18S forward: CGGCTACCACATCCAAGGAA; reverse: GCTGGAATTACCGCGGCT and NOS3: forward: ATGGCGAAGCGAGTGAAG; reverse: ACTCATCCATACACAGGACCC. Relative expression levels were calculated using the ΔΔCt method. Expression of NOS3 mRNA was normalised to the average of the untreated control and, as is standard practice for qPCR data, expressed as relative fold change. Untreated control samples retained their individual fold change. For qPCR experiments, a one‐way ANOVA with Tukey's post test was performed to compare relative fold change between treatment groups.

### 2.7 Regulation of endothelial cell proliferation

Apelin has been reported as a pro‐proliferative and anti‐apoptotic factor in endothelial cells (Alastalo et al., 2011; Kim et al., 2013). We have tested if MM07 could exert this effect in human PAECs. To study cell proliferation, human PAECs (Lonza, passages 4, *n* = 6 independent experiments) were seeded at 50,000 cells per well in 8‐well chamber slides (BD Falcon, Bedford, MA, USA) in EGM‐2 with 10% FBS, allowed to attach, and then starved overnight in EBM‐2 with 0.5% FBS to inhibit cell division. The cells were then treated for 24 hr in EBM‐2 with 2% FBS with PBS, [Pyr^1^]apelin‐13 (1 μM), MM07 (10 μM), or recombinant human VEGF (rhVEGF, 20 ng·ml^−1^, cat. no. 293‐VE, R&D Systems, Minneapolis, MN, USA) as a positive control. The treatment media also contained 20μM 5‐ethynyl‐2′‐deoxyuridine (EdU, part of the Click‐iT® EdU Imaging Kit, cat. no. C10337, Invitrogen) as a nucleoside analogue incorporated in DNA as the cells replicate. Detection of the incorporated EdU was carried out following the manufacturer's instructions. The cells were fixed with 4% paraformaldehyde with 0.5% Triton‐X for 20 min and stained with Click‐iT EdU reaction buffer containing AlexaFluor 488 azide, copper sulphate, and Click‐iT EdU buffer additive for 30 min at room temperature. The chambers were mounted in DAPI Vectashield medium (Vectorlabs, Peterborough, UK) and imaged for blue and green fluorescence on an Olympus BX63 microscope with an EXi Blue camera (QImaging, Surrey, BC, Canada) using the cellSens software (RRID:SCR_016238, v1.14, Olympus, Southend‐on‐Sea, UK). For each cell chamber, images were taken at 10× magnification for five random fields based on blue DAPI fluorescence. Counting of DAPI and EdU positive cells were performed using the cellSens software with a cell area threshold to exclude cell debris and doublets applied to all images. The amount of cell proliferation was measured as the percentage of EdU positive cells to DAPI positive cells.

### 2.8 Rescue of endothelial cell apoptosis

The effects of MM07 on endothelial cell apoptosis was tested and compared with those of rhVEGF, using human PAECs (Lonza, passages 4–6, *n* = 3 donors with three independent experiments each) as previously described (Long et al., 2015) following protocol optimisation in donor lines 1–3. Briefly, PAECs were seeded in six‐well tissue culture plates at 200,000 cells·per well in EGM‐2 with 10% FBS and allowed to attach. On the next day, wells were washed with PBS, and the media changed to either EBM‐2 with 2% FBS or 10% FBS controls. MM07 (10 μM) or rhVEGF (10 ng·ml^−1^) were added to the wells and incubated for 18 hr. Apoptosis was induced by incubating the cells with TNF‐α (cat. no. 210‐TA, R&D Systems; 1.5 ng·ml^−1^) and cycloheximide (CHX; cat. no. c7698, σ, 20 μg·ml^−1^) for 5 hr in the experimental wells. Control wells did not receive TNF‐α and CHX Treatment. Cells were then washed in PBS, trypsinised (Lonza), transferred into binding buffer for the apoptosis assay (cat. no. v13242, Thermofisher/Invitrogen) and stained with anti‐annexin FITC‐conjugated antibody (1:2 stock dilution) and propidium iodide (PI, 20 μg·ml^−1^) for 15 min at room temperature. Cells were filtered through 50‐μm filters (Sysmex Partek, Goerlitz, Germany) and kept on ice before flow cytometry (BD Canto II, BD Biosciences, San Jose, CA, USA). For each condition, 10,000 events were recorded. Data analysis was performed on FlowJo v10 (RRID:SCR_008520, FlowJo LLC, Oregon, USA). Annexin^+^/PI^+^ cells were classified as “dead,” Annexin^+^/PI^−^ cells “apoptotic” and Annexin^−^/PI^−^ as “healthy.” Gates were adjusted such that approximately equal numbers of “healthy” and “apoptotic” cells occurred in the TNF‐α/CHX treated group, as this provided a large window for either further induction or rescue of apoptosis. The raw percentage of cells in each gate were used in the data analysis. For statistical analysis, technical replicates were excluded if the apoptotic induction was less than 5%. The average induction of apoptosis of the analysed experiments was 19.53 ± 1.76%.

### 2.9 Data and statistical analysis

The data and statistical analysis comply with the recommendations of the *British Journal of Pharmacology* on experimental design and analysis in pharmacology. Data are expressed as mean ± SEM. Statistical analyses were conducted in GraphPad Prism 6 (RRID:SCR_002798). Post hoc tests were carried out only if F was significant and there was no variance in homogeneity. The number of animals in each group included for statistical tests is shown in the figure legend for that analysis. For the human PAEC assays except qPCR, as some variability in the basal amount of eNOS phosphorylation, cell proliferation, and apoptotic induction was observed, as illustrated by the example of variable amounts of basal apoptotic response (Figure S1). To minimise the confounding variability between independent experiments, a repeated measures one‐way ANOVA with Dunnett's post test was performed to compared treated groups with PBS‐treated control group. For all experiments, *P* ≤ 0.05 was considered statistically significant.

### 2.10 Materials

All chemical reagents were purchased from Sigma (Poole, UK), unless otherwise stated. Human [Pyr^1^]apelin‐13 and MM07 were synthesised by Severn Biotech (Kidderminster, UK) to GLP standard using Fmoc chemistry on a solid phase support matrix to 98% purity by Maldi‐TOF mass spectroscopy and HPLC analysis. Peptides were tested for sterility and demonstrated to be pyrogen free, and biological activity was confirmed using a β‐arrestin recruitment assay (DiscoverX, Fremont, USA) as described (Yang, Read, et al., 2017).

### 2.11 Nomenclature of targets and ligands

Key protein targets and ligands in this article are hyperlinked to corresponding entries in http://www.guidetopharmacology.org, the common portal for data from the IUPHAR/BPS Guide to PHARMACOLOGY (Harding et al., 2018), and are permanently archived in the Concise Guide to PHARMACOLOGY 2017/18 (Alexander, Cidlowski et al., 2017; Alexander, Christopoulos et al., 2017; Alexander, Fabbro et al. 2017a, 2017b).

## 3 RESULTS

### 3.1 Cardioprotective effects of MM07 shown by cardiac MRI

**Figure 2:**
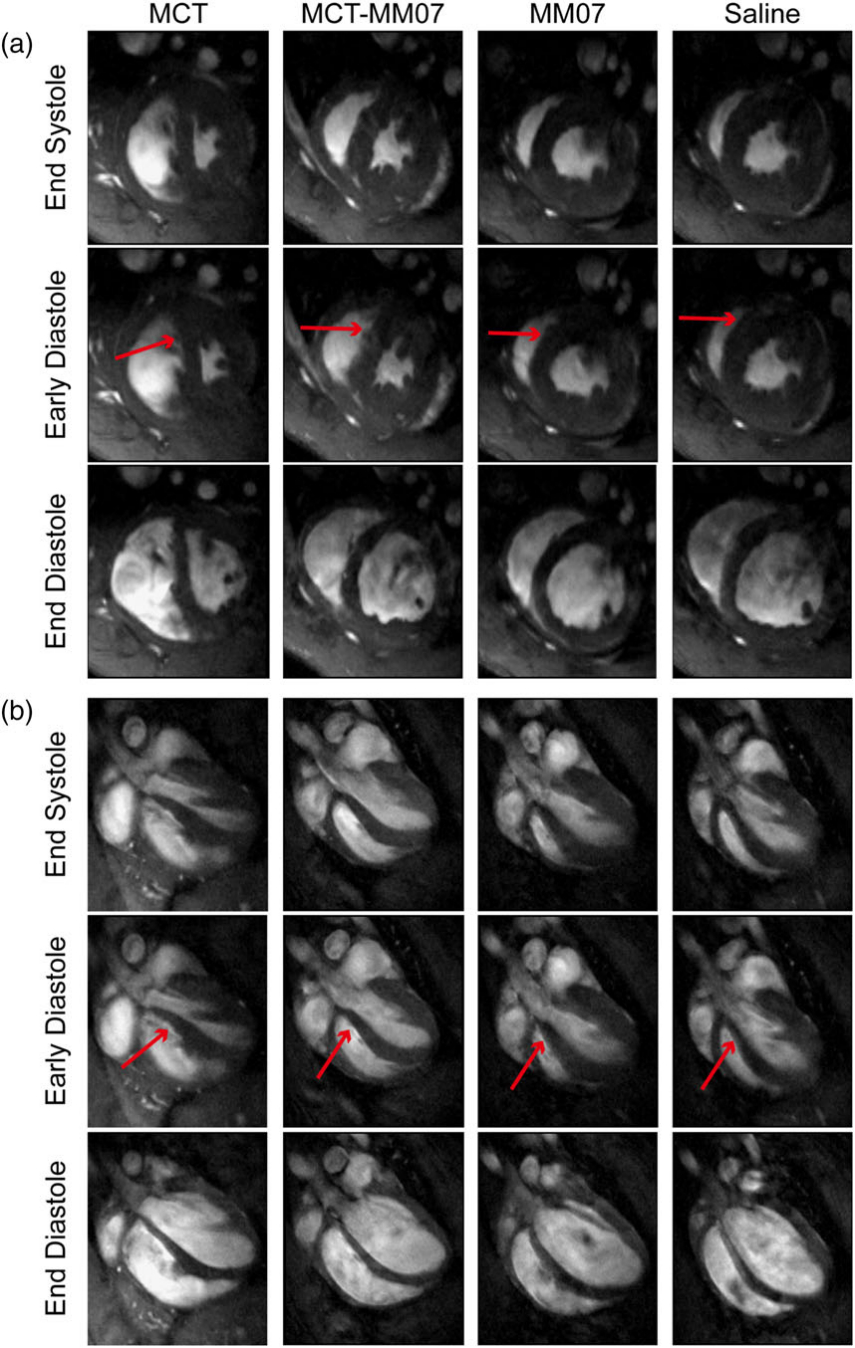
MRI of rat hearts in the monocrotaline (MCT)‐MM07 study. Representative snapshots of cardiac MRI scans at end‐systolic, early diastolic, and end‐diastolic points from MCT, MCT + MM07, MM07, and saline‐injected rats. (a) Short‐axis views with the right ventricle on the left side. (b) Long‐axis views with the right ventricle at the bottom. Red arrows are drawn to show the point of septal wall bowing at early diastole

On Day 20, changes in the appearance and motion of ventricles were detectable by non‐invasive cardiac MRI, as shown in the snapshots of the heart of representative animals in the short‐ (Figure 2a) and long‐axis (Figure 2b) views and in the videos (Videos 1–8). Compared to the saline‐injected control rats, MCT‐exposed rats developed an enlarged RV. At the end of systole, MCT‐exposed rats had more residual volume in the RV compared to the controls. In early diastole, MCT exposure resulted in a distortion in the interventricular septal wall. The filling of the LV and RV appeared to be less synchronous in the MCT‐exposed animals, and the LV appeared to be insufficiently filled at the end of diastole. Administration of MM07 to MCT‐exposed rats attenuated these structural changes, as their heart appeared to be intermediate between MCT and saline control animals. Additionally, MM07 alone did not appear to alter cardiac structure and function, compared with the saline controls.

**Figure 3:**
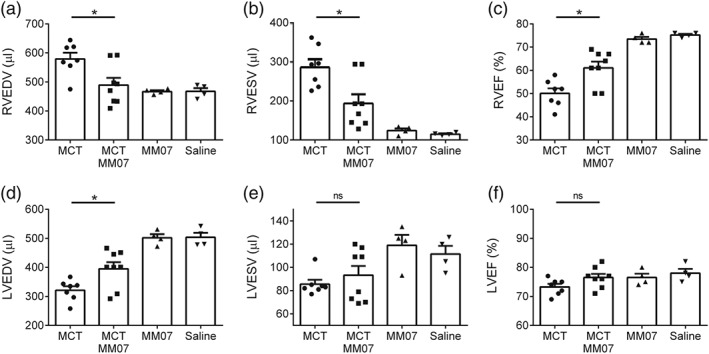
Quantitative analysis of cardiac MRI. (a) MM07 attenuated monocrotaline (MCT)‐induced increase in right ventricular end‐diastolic volume (RVEDV). (b) MM07 attenuated MCT‐induced increase in right ventricular end‐systolic volume (RVESV). (c) MM07 attenuated MCT‐induced decrease in right ventricular ejection fraction (RVEF). (d) MM07 attenuated MCT‐induced decrease in left ventricular end‐diastolic volume (LVEDV). (e) MCT and MM07 did not alter left ventricular end‐systolic volume (LVESV). (f) MCT and MM07 did not alter left ventricular ejection fraction (LVEF). (MCT *n* = 7, MCT‐MM07 *n* = 8, MM07 *n* = 4, and saline *n* = 4). Data shown are individual values with means ± SEM. *P ≤ 0.05, significantly different; ns: not significant, as indicated; Student's two‐tailed *t* test

Quantitative analysis of the cardiac MRI revealed that MCT exposure resulted in an increase in RV end‐diastolic and end‐systolic volumes (Figure 3a,b), indicating an enlarged RV that could not contract to empty sufficiently. The combined effect of these was a reduced RV ejection fraction, compared with that in the saline‐injected control rats (Figure 3c). MCT exposure also caused LV underfilling as a consequence of reduced LV end‐diastolic volume (Figure 3d), without altering LV end‐systolic volume and ejection fraction (Figure 3e–f). MM07 treatment significantly attenuated MCT‐induced changes in RV end‐diastolic and end‐systolic volumes, ejection fraction, and LV end‐diastolic volume. In the rats not exposed to MCT, all measured parameters with MM07 alone were comparable to the saline controls (Figure 3a–f).

### 3.2 MM07 attenuated the development of PAH in MCT‐exposed rats

**Figure 4:**
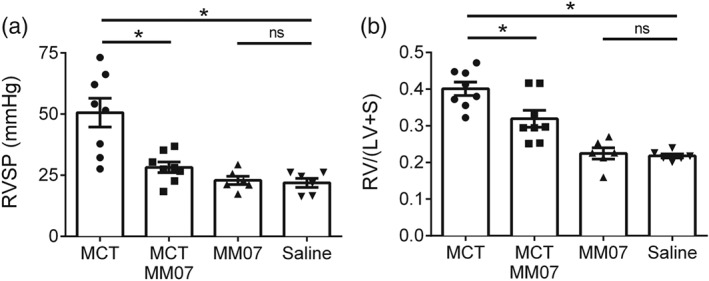
Attenuation of monocrotaline (MCT)‐induced pulmonary arterial hypertension by MM07 administration. (a) MM07 attenuated MCT‐induced elevation in right ventricular systolic pressure (RVSP). (b) MM07 attenuated MCT‐induced right ventricular (RV) hypertrophy, measured as RV/(LV + S), or Fulton index (MCT *n* = 8, MCT‐MM07 *n* = 8, MM07 *n* = 6, and saline *n* = 6). Data shown are individual values with means ± SEM. *P ≤ 0.05, significantly different; ns: not significant, as indicated; one‐way ANOVA with Tukey's post test. LV: left ventricle; S: septum

Next, invasive haemodynamic measurements were carried out in isoflurane‐anaesthetised rats. Compared with the saline‐injected rats, RVSP was significantly elevated in MCT‐exposed rats (Figure 4a; Table 1), indicating that these animals have developed pulmonary hypertension as expected. Ccompared with this group, RVSP was significantly lower in MCT‐exposed rats treated with MM07, to a level that is higher than but not statistically different from the saline‐injected controls. MM07 injection alone did not alter RVSP compared to the vehicle‐injected group.

**Table 1:** MM07 attenuated MCT‐induced changes in RVSP, RV hypertrophy, and pulmonary vascular remodelling

	MCT	MCT + MM07	MM07	Control
*n* (number)	8	8	6	6
Body weight (g)	303 ± 8	295 ± 4	343 ± 9	343 ± 2
RVSP (mmHg)	50.6 ± 5.9*	28.2 ± 2.2†	22.9 ± 1.7	21.9 ± 1.9
Systolic pressure (mmHg)	105.5 ± 2.0	105.2 ± 1.2	108.2 ± 3.7	104.5 ± 1.6
Diastolic pressure (mmHg)	76.5 ± 2.5	76.4 ± 0.9	77.0 ± 2.0	77.3 ± 1.2
Heart rate (bpm)	363 ± 6	365 ± 7	368 ± 15	361 ± 15
RV weight (g)	0.27 ± 0.02	0.23 ± 0.02	0.16 ± 0.01	0.16 ± 0.01
LV + S weight (g)	0.67 ± 0.02	0.70 ± 0.02	0.71 ± 0.02	0.74 ± 0.01
RV/(LV + S)	0.40 ± 0.02*	0.33 ± 0.03* †	0.22 ± 0.02	0.21 ± 0.01
Fully muscularised vessels (%)	32 ± 1*	24 ± 2* †	15 ± 1	14 ± 1
Vessel wall thickness (%)	21 ± 1*	16 ± 1* †	10 ± 1	10 ± 1

Results shown are from rats at the end of the experiments (Day 21). Data are expressed as mean ± SEM.

* *P* ≤ 0.05, significantly different from Control (saline‐treated);

† *P* ≤ 0.05, significantly different from MCT‐exposed rats; one‐way ANOVA with Tukey's post test. LV, left ventricle; MCT, monocrotaline; RV, right ventricle; RVSP, right ventricular systolic pressure; S, septum.

### 3.3 MM07 attenuated MCT‐induced RV hypertrophy

After the animals were killed, structural changes in heart ventricles were assessed. Compared with the controls, the Fulton index were significantly increased in in MCT‐exposed rats, indicating the development of RV hypertrophy (Figure 4b; Table 1). This structural change of the RV was significantly attenuated by MM07 administration. However, MM07 did not completely prevent the development of RV hypertrophy resulting from MCT. MM07 injection alone did not alter the Fulton index, compared with the saline controls.

### 3.4 MM07 attenuated MCT‐induced pulmonary vascular remodelling

**Figure 5:**
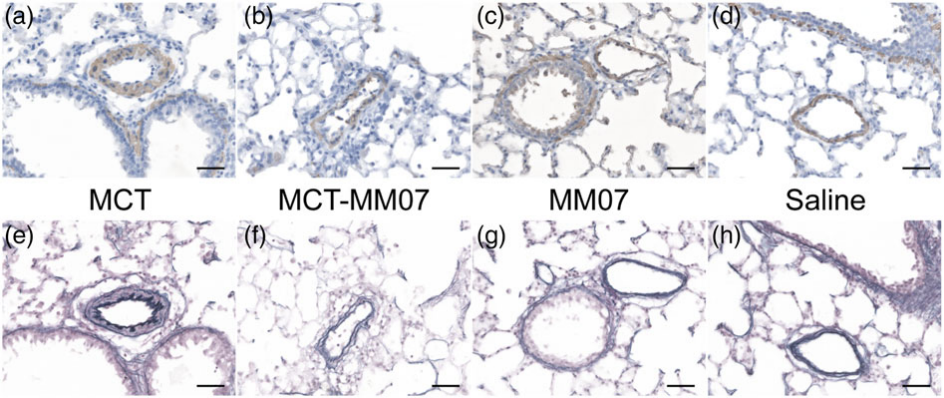
Immunohistological visualisation of remodelling of pulmonary arterioles. Results shown are from a vessel close to a terminal bronchiole, using α‐smooth muscle actin ([a–d] brown colour) and (e–h) van Gieson's stain, in sections of lung from (a,e) MCT, (b,f) MCT + MM07, (c,g) MM07, and (d,h) saline control rats (scale bars = 75 μm)

**Figure 6:**
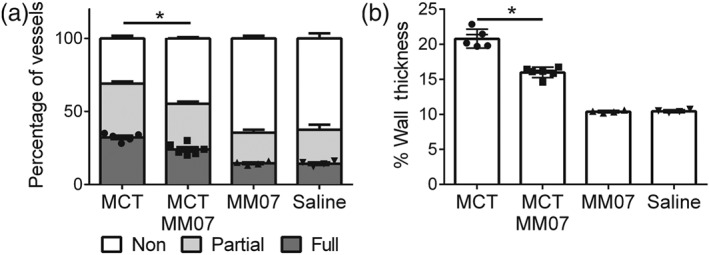
Attenuation of monocrotaline (MCT)‐induced pulmonary vascular remodelling by MM07 administration (MCT *n* = 5, MCT‐MM07 *n* = 6, MM07 *n* = 4, and saline *n* = 4). Results are expressed as (a) proportion of muscularised vessels in rat lung and (b) wall thickness of larger, fully muscularised pulmonary arterioles. For (a), statistical significance was assessed by comparing the percentage of fully muscularised vessels between groups. For MCT‐treated animals, the effects of MM07 and saline were compared. Data shown are individual values with means ± SEM. *P ≤ 0.05, significantly different as indicated; Student's two‐tailed *t* test

In order to determine the effect of MM07 on the pathogenesis of PAH, morphometry of pulmonary arterioles was examined, and the extent of vascular remodelling was scored. Representative photomicrographs of rat lung sections with smooth muscle actin and van Giesen's staining are shown in Figure 5. As expected, MCT injection increased the percentage of fully muscularised small vessels (Figure 6a) and the wall thickness of small to medium‐sized arterioles (Figure 6b; Table 1). MCT‐induced vascular remodelling was attenuated by MM07 administration, as the proportion of fully muscularised small vessels and the arteriolar wall thickness were significantly reduced. The extent of small vessel muscularisation and arteriolar wall thickness was similar in saline and MM07‐injected control groups.

### 3.5 MM07 activates eNOS and AMPK

**Figure 7:**
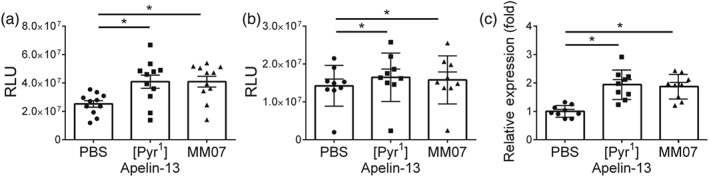
MM07 activated eNOS and AMPK. (a) Stimulation of human pulmonary artery endothelial cells (PAECs) with [Pyr^1^]apelin‐13 or MM07 increased the level of phosphorylated eNOS measured by chemiluminescence (*n* = 11 each). Data shown are individual values with means ± SEM. *P ≤ 0.05, significantly different as indicated; one‐way ANOVA with Tukey's post test. (b) Stimulation of human PAECs with [Pyr^1^]apelin‐13 or MM07 increased the level of phosphorylated AMPKa measured by chemiluminescence (*n* = 11 each). RLU: relative light units. Data shown are individual values with means ± SEM. *P ≤ 0.05, significantly different as indicated; repeated measures ANOVA with Dunnett's post test. (c) Stimulation of human PAECs with [Pyr^1^]apelin‐13 or MM07 increased the level of mRNA of eNOS. Data shown are individual values with means ± SEM. *P ≤ 0.05, significantly different as indicated; one‐way ANOVA with Tukey's post test

In order to investigate the potential mechanisms underlying the benefit of MM07 in MCT‐induced PAH, we have tested the effect of MM07 on protective signalling in PAECs known to be activated by apelin signalling. Knockdown of apelin expression reduces the phosphorylation and activation of eNOS and AMPK in PAECs (Chandra et al., 2011). Human PAECs were stimulated acutely with [Pyr^1^]apelin‐13 or MM07. Both ligands significantly increased phosphorylation of eNOS (Figure 7a) and AMPKa (Figure 7b), compared with control cells. Stimulation of PAECs with [Pyr^1^]apelin‐13 or MM07 also increased the mRNA levels of eNOS (NOS3 gene; Figure 7c).

### 3.6 MM07 promoted proliferation of pulmonary arterial endothelial cells

**Figure 8:**
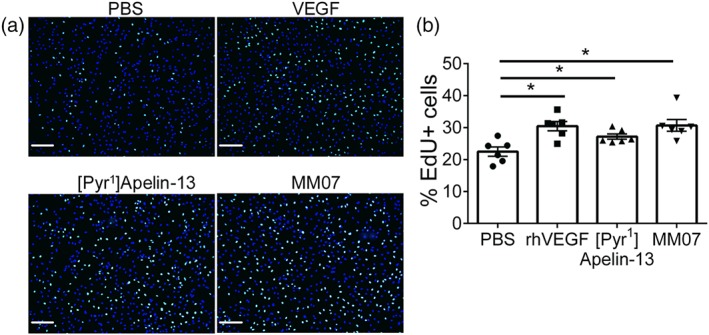
MM07 promoted proliferation of pulmonary arterial endothelial cells. (a) Representative two‐channel overlay photomicrographs of cells treated with PBS (baseline), recombinant human VEGF (rhVEGF; as a positive control), [Pyr^1^]apelin‐13, or MM07, showing EdU negative cell nuclei in blue and EdU positive nuclei in cyan. Scale bar = 200 μm. (b) Stimulation of human pulmonary artery endothelial cells with rhVEGF, [Pyr^1^]apelin‐13, or MM07 increased the proportion of EdU positive cells (*n* = 6 each). Data shown are individual values with means ± SEM. *P ≤ 0.05, significantly different as indicated; repeated measures ANOVA

Next, we examined the functional effect of MM07 on endothelial cell homeostasis. Previous studies have shown that apelin induced proliferation of PAECs and PMVECs (Alastalo et al., 2011; Kim et al., 2013). We have stimulated human PAECs with ligands and measured cell proliferation using a DNA replication‐based assay. Significantly, more EdU incorporation was induced by the positive control treatment, rhVEGF, compared with untreated controls. Similarly, both [Pyr^1^]apelin‐13 and MM07 induced a higher proportion of proliferating cells (Figure 8a for representative images and 8b for inter‐group comparisons).

### 3.7 MM07 exerted anti‐apoptotic effects on pulmonary arterial endothelial cells

**Figure 9:**
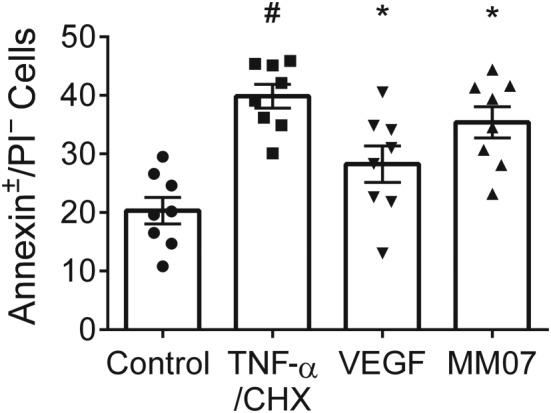
Rescue of human pulmonary arterial endothelial cells from apoptosis induced by TNF‐α/cycloheximide (CHX; *n* = 8 independent experiments using cells from three donors). TNF‐α/CHX significantly induced apoptosis, relative to the endothelial basal medium 2 2% FBS serum and growth factor starved control. Recombinant human VEGF (rhVEGF) pretreatment for 18 hr significantly attenuated this apoptotic induction as did MM07. Data shown are individual values with means ± SEM. ^#^ *P* < 0.05, significantly different from control; *P ≤ 0.05, significantly different from TNF‐α/CHX; repeated measures ANOVA

Apelin has been reported to have anti‐apoptotic roles on endothelial cells in the pulmonary circulation (Alastalo et al., 2011; Kim et al., 2013). The effect of MM07 on apoptosis of primary human PAECs was investigated using donor cell lines. Treatment with TNF‐α/CHX increased Annexin^+^/PI^−^ staining (19.5%). Pretreatment with rhVEGF for 18 hr significantly attenuated this apoptotic induction (11.6%) as did MM07 (4.5%; Figure 9). In control experiments, EBM‐2 2% FBS control (serum and growth factor starved) was compared with a healthy control (EGM‐2 10% FBS) and the extent of rescue by rhVEGF or MM07, following serum and growth factor starvation, assessed. Serum and growth factor starvation significantly induced Annexin^+^/PI^−^ staining (9.3%), which was almost wholly rescued by 18 hr of rhVEGF pretreatment (8.9%). However, pretreatment with MM07 did not rescue the cells from serum and growth factor starvation (Figure S2).

## 4 DISCUSSION AND CONCLUSIONS

This is the first study of an apelin receptor biased agonist in an animal model of disease. Our results showed that MM07 attenuated the elevation of RVSP, RV hypertrophy, cardiac dysfunction, and pulmonary vascular remodelling induced by MCT. MM07 also exerted pro‐proliferative and anti‐apoptotic effects on human PAECs in vitro. Our findings suggest that G protein‐biased agonism of the apelin receptor has potentially beneficial effects in PAH, indicating that the apelin receptor is a therapeutic target, responsive to biased agonism.

The goal of supplying exogenous apelin receptor agonist in PAH is to replace the missing endogenous peptide. Reduced apelin expression results from the dysfunction of bone morphogenetic protein receptor type 2 (Alastalo et al., 2011), which is the most frequently mutated gene in familial and sporadic PAH (Machado et al., 2015) and a critical component in a pathway central to PAH pathogenesis (Morrell et al., 2016). It has been reported that apelin is a transcriptional target of bone morphogenetic protein receptor type 2, mediated by the miR‐130/301 family and PPARγ in pulmonary vascular endothelial cells (Alastalo et al., 2011; Bertero et al., 2014). Furthermore, apelin expression in the RV is decreased in the MCT (Falcão‐Pires et al., 2009) and Sugen 5416 + hypoxia‐induced (Drake et al., 2011; Frump et al., 2015) rat models of PAH. Importantly, apelin levels were correlated with contractile and diastolic function of the RV in the Sugen + hypoxia model (Neto‐Neves, Frump, Vayl, Kline, & Lahm, 2017). Interestingly, elabela/toddler, the second endogenous ligand of the apelin receptor encoded by *APELA* gene, is also reduced in cardiopulmonary tissues from PAH patients and animal models (Yang, Read, et al., 2017). In an early study of particular interest, the apelin peptide was tested in MCT‐induced PAH (Falcão‐Pires et al., 2009). The apelin signalling pathway is also implicated in pulmonary veno‐occlusive disease (Lathen et al., 2014) and chronic thromboembolic pulmonary hypertension (Miao et al., 2017). As expected, agents that activate apelin expression or act as downstream effectors of apelin signalling have demonstrated beneficial effects in PAH animal models (Bertero et al., 2014; Kim et al., 2013; Nickel et al., 2015; Spiekerkoetter et al., 2013).

One caveat may limit the therapeutic efficacy and clinical development of native apelin peptides. As a GPCR, the ligand‐activated apelin receptor is phosphorylated by GPCR kinases, and β‐arrestin is recruited to the receptor. This switches off G protein‐mediated signalling and triggers receptor desensitisation, internalisation, and removal of the bound apelin ligand, leading to loss of efficacy with chronic administration of the peptide (Evans et al., 2001; Rajagopal, Rajagopal, & Lefkowitz, 2010; Violin & Lefkowitz, 2007). Importantly, β‐arrestin‐mediated signalling is also involved in an apelin‐independent, stretch‐sensitive function of the apelin receptor and may cause myocardial hypertrophy and heart failure, whereas apelin‐induced G_αi_ signalling is protective (Scimia et al., 2012). This need to selectively or preferentially activate a subset of downstream signalling pathways forms the rationale to investigate signalling bias and develop G protein‐biased agonists of the apelin receptor. Using a number of in vitro screening assays, our group identified MM07 as the first apelin analogue peptide, acting as a G protein‐biased agonist of the apelin receptor (Brame et al., 2015).

Having demonstrated the activity of MM07 in vivo under normal physiological conditions (Brame et al., 2015), we tested this peptide or, more generally, the concept of G protein‐biased agonism of the apelin receptor, in a disease model. The current study used an experimental design with the same dose of MCT and time course as Falcão‐Pires et al. (2009), in order to confirm that MM07 could attenuate the development of PAH. In this previous 3‐week experiment, rats injected with 60 mg·kg^−1^ MCT were treated with i.p. injections of the predominant endogenous apelin peptide, [Pyr^1^]apelin‐13, at a dose of 200 μg·kg^−1^·day^−1^. The authors reported that [Pyr^1^]apelin‐13 ameliorated the elevation in RVSP and development of RV hypertrophy but failed to attenuate the thickening of pulmonary arterioles (Falcão‐Pires et al., 2009). Owing to the lower receptor binding affinity and potency of MM07 compared with that of [Pyr^1^]apelin‐13 (Brame et al., 2015), a higher dose of MM07 (1 mg·kg^−1^·day) was used compared to the previous study with [Pyr^1^]apelin‐13 (200 μg·kg^−1^·day^−1^ in Falcão‐Pires et al., 2009).

The cardiac MRI showed that MCT caused an enlargement of the RV and systolic dysfunction, indicating the RV failed to match the increased afterload due to increased pulmonary vascular resistance, consistent with previous reports (Porvasnik et al., 2010; Redout et al., 2010). The combined effect of a lowered RV output, compression of the LV due to RV enlargement, and the leftward septal displacement led to underfilling of the LV, as shown by the reduced LV volume at end diastole (Gan et al., 2006). Importantly, MM07 attenuated the detrimental effects of MCT by limiting the enlargement of the RV and improving its ejective function, which in turn alleviated underfilling of the LV. In healthy control animals, MM07 did not significantly alter any of these parameters, suggesting that its actions were only manifest following injury with MCT and that MM07 did not affect the normal physiological functions of the pulmonary circulation. Previous studies have proposed possible explanations for the observed benefits of MM07. Apelin receptor signalling is known to increase cardiac contractility and output (Brame et al., 2015; Maguire et al., 2009; Szokodi et al., 2002), possibly by activating myosin light chain kinase to increase myofilament calcium sensitivity (Perjés et al., 2014) and by a positive lusitropic effect (Peyronnet et al., 2017), so the enhanced cardiac performance does not require an increase in oxygen consumption (Charo et al., 2009). MM07‐induced haemodynamic improvement was confirmed by invasive right heart catheterisation. MM07‐injected rats were shown to have significantly lower RVSP in response to MCT exposure, compared with those not treated with MM07. This is unlikely to be caused by an acute vasodilatory effect of MM07, as the compound was not injected on the day of catheterisation. In agreement, chronic administration of the native apelin peptide reduced RVSP in mouse and rat models of PAH (Alastalo et al., 2011; Falcão‐Pires et al., 2009). Importantly, MCT‐induced RV hypertrophy was less pronounced in MM07‐treated rats, consistent with the previous study showing that apelin receptor ligand‐activated G_αi_ protein signalling protected against cardiac hypertrophy and failure (Scimia et al., 2012). Taken together, our physiological results have demonstrated protective effects of MM07 against MCT‐induced cardiac dysfunction.

While the signalling pathways activated by the apelin receptor are not fully characterised, previous studies have shown that eNOS expression and phosphorylation are reduced with apelin knockdown and in apelin knockout animals (Chandra et al., 2011), but eNOS phosphorylation can be induced by [Pyr^1^]apelin‐13 and elabela/toddler (Yang, Read, et al., 2017). We have found that MM07 could also induce eNOS phosphorylation acutely and then increase eNOS expression in human PAECs. The important role of eNOS is demonstrated by the increased susceptibility to hypoxia‐induced pulmonary hypertension in eNOS deficient mice (Fagan et al., 1999) and by the therapeutic benefit of eNOS gene transfer in MCT‐exposed rats (Zhao et al., 2006). Importantly, eNOS functions beyond generating a vasodilator, as eNOS gene transfer restored the microvascular loss in the MCT model (Zhao et al., 2006). AMPK mediated the pro‐angiogenic effects of apelin and activation of AMPK has beneficial effects in PAH animal models (Chandra et al., 2011; Yang et al., 2014). We have found that MM07 could increase the phosphorylation of AMPKa in endothelial cells. There are other potential mechanisms for the beneficial effects of MM07, as it is clear that apelin receptor signalling regulates multiple effector pathways and cell types in the pulmonary vasculature. For example, apelin was reported to induce the endothelial ATPase CD39 and limit the emergence of apoptosis‐resistant PAECs (Helenius et al., 2015), possibly at a later stage of PAH pathogenesis. Moreover, it has been reported that apelin regulates myocyte enhancer factor 2, which in turn activates miR‐424/miR‐503 and genes contributing to endothelial homeostasis. This axis also inhibits the expression of FGF‐2 and its receptor and thus exerts anti‐proliferative effects on pulmonary arterial smooth muscle cells (Alastalo et al., 2011; Helenius et al., 2015; Kim et al., 2013, 2015). Further experiments, possibly using a co‐culture system, are required to understand how G protein‐biased apelin signalling in endothelial cell may affect the homeostasis of smooth muscle cells.

The benefits of apelin may extend beyond its acute vasodilatory and positive inotropic activities, as apelin regulates the homeostasis of pulmonary vascular cells. Conversely, deficiency of apelin may contribute to the imbalance between proliferation and apoptosis of these cells, leading to pathological vascular remodelling, which is the ultimate cause of PAH (Andersen, Hilberg, Mellemkjaer, Nielsen‐Kudsk, & Simonsen, 2011; Yang et al., 2015). In the current study, MM07 demonstrated disease modification, as it attenuated pulmonary vascular remodelling in terms of arteriolar muscularisation and wall thickening, thereby mitigating the key driving feature in the pathogenesis of PAH (Morrell et al., 2016; Rabinovitch, 2012). Endothelial apoptosis is thought to initiate PAH and contribute to the loss of microvasculature in human disease and animal models (Rabinovitch, 2012; Wilson et al., 1992). In this study, we have investigated the effect of MM07 on the homeostasis of PAECs, in order to understand the mechanism underlying its protective effects on vascular remodelling. Apelin promoted the survival of pulmonary vascular endothelial cells (Alastalo et al., 2011; Kim et al., 2013) and we have used an EdU incorporation assay to measure cell proliferation, which has the advantage of not being confounded by the metabolic state of the cell, as it measures DNA synthesis directly. We found that MM07 promoted PAEC proliferation in 24 hr, as did [Pyr^1^]apelin‐13. To test the ability of MM07 to rescue endothelial apoptosis, we utilised a previously published apoptosis assay using TNF‐α/CHX to induce apoptosis in healthy human PAECs (Long et al., 2015). Under normal conditions, TNF‐α prevents apoptosis of endothelial cells through activation of the NF‐κB pathway whereas, in conditions of global protein synthesis suppression, such as produced by concurrent application of CHX, apoptosis is induced through TNF‐R1 leading to JNK phosphorylation. In our experiments, MM07 reduced apoptosis of healthy human PAECs induced by TNF‐α/CHX, demonstrating prevention of endothelial damage in vitro. Interestingly, MM07 did not rescue endothelial damage caused by growth factor and serum starvation, a less severe treatment than TNF‐α/CHX, suggesting it may be more suitable in early disease where high levels of endothelial damage are thought to be critical drivers of disease development. We acknowledge that PMVECs may be closer to the endothelium from the small pulmonary vessels, than PAECs. However, the pro‐proliferative and anti‐apoptotic effects of native apelin peptide have been reported in both normal PAECs and PMVECs (Alastalo et al., 2011; Kim et al., 2013) and even in endothelial cells from other vascular beds or organisms (see Pitkin, Maguire, Bonner, & Davenport, 2011). Overall, our finding provides a mechanism and supports apelin mimetics as important in treating disease aetiology itself, rather than simply relying on vasodilatation, as is the case with currently used therapeutic agents.

Recent studies have generated more insight on the apelin receptor structure (Ma et al., 2017), apelin degradative pathways (McKinnie et al., 2016; Murza, Belleville, Longpre, Sarret, & Marsault, 2014; Yang, Kuc, et al., 2017), antagonists (Le Gonidec et al., 2017), and more peptide biased agonists of the apelin receptor (Ceraudo et al., 2014; McAnally et al., 2017; Murza et al., 2017), providing new tools to further investigate biased agonism of the apelin receptor. One property that distinguishes MM07 from [Pyr^1^]apelin‐13 is pharmacokinetic stability. Although still relatively short at under 20 min, MM07 has a significantly longer in vivo plasma half‐life than [Pyr^1^]apelin‐13 (Brame et al., 2015), which may be a consequence of its N terminal cyclisation and/or reduced removal due to receptor internalisation. This may have enabled MM07 to produce greater target cover compared with [Pyr^1^]apelin‐13 with each daily injection. Our previous human study showed that the magnitude of vascular response decreased with repeated dosing of [Pyr^1^]apelin‐13, but not with MM07, because of receptor desensitisation (Brame et al., 2015). This phenomenon may also contribute to the benefit of MM07 observed in the current chronic study. Building on the efficacy of MM07, novel biased apelin receptor agonists with higher binding affinity, higher potency in G protein signalling, more G protein bias, and even better stability, such as the low MW compound CMF‐019 (Read et al., 2016), could be tested in PAH animal models in future studies. As G protein‐biased agonism of the apelin receptor was shown to be safe in chronic use, further studies may be able to reduce the number of animals in the agonist‐only control group.

This study has demonstrated that a G protein‐biased agonist of the apelin receptor could attenuate the development of MCT‐induced PAH, which is an acute model of moderate severity. In future studies, this concept could be validated in the Sugen5416 and chronic hypoxia‐induced model of PAH, which exhibits more severe pulmonary vascular remodelling (Stenmark, Meyrick, Galie, Mooi, & McMurtry, 2009). More importantly, what is required to corroborate the data we have obtained with MM07 in the MCT prevention model is testing of an apelin G protein‐biased agonist in a more clinically relevant, reversal model of PAH. This may require the development of apelin agonists with improved pharmacokinetic properties to allow maintained target exposure from once a day administration beyond that which is likely achieved with the relatively short‐lived peptide MM07.

In conclusion, this proof‐of‐principle study demonstrated the effectiveness of MM07, a G protein‐biased agonist of the apelin receptor, in attenuating the development of MCT‐induced PAH. In addition to reducing the elevation of RVSP and hypertrophy as previously shown with [Pyr^1^]apelin‐13, MM07 also attenuated functional changes of the heart and vascular remodelling in the lung. At the cellular level, MM07 acts as a pro‐proliferative and anti‐apoptotic factor that activates beneficial signalling in endothelial cells. By selectively activating downstream pathways, biased agonists have been shown to be as effective as the native unbiased agonist with additional benefits, reflecting the differential signalling. This is illustrated by the G protein‐biased agonists of the μ‐opioid receptor, TRV130 (oliceridine) and PZM21, which produced greater pain relief than morphine with reduced β‐arrestin‐mediated respiratory depression (Manglik et al., 2016; Viscusi et al., 2016). The results from the current study suggest that biased agonism may also be exploited in the treatment of PAH.

## CONFLICT OF INTEREST

The authors declare no conflicts of interest.

## AUTHOR CONTRIBUTIONS

P.Y., C.R., R.E.K., D.N., T.L.W., A.C., G.B., M.S., S.J.S., N.W.M., A.P.D., and J.J.M. have made substantial contributions to conception and design or acquisition of data or analysis and interpretation of data. R.C.G. design of MM07 P.Y., C.R., A.P.D., and J.J.M. were involved in drafting the manuscript or revising it critically for important intellectual content.

## DECLARATION OF TRANSPARENCY AND SCIENTIFIC RIGOUR

This Declaration acknowledges that this paper adheres to the principles for transparent reporting and scientific rigour of preclinical research as stated in the *BJP* guidelines for Design & Analysis, Immunoblotting and Immunochemistry, and Animal Experimentation, and as recommended by funding agencies, publishers and other organisations engaged with supporting research.

## Supporting information

Figure S1: Annexin+/PI‐ stained cells in each condition for each experimental replicate. Staining was consistent throughout experiments as indicated by the limited crossover between the lines, however, basal staining varied between experiments. A matched ANOVA was used so that data trends could be assessed.
Figure S2: Rescue of apoptosis of human pulmonary arterial endothelial cells induced by serum and growth factor starvation. Serum and growth factor starvation significantly induced apoptosis relative to the EGM‐2 10%FBS control (9.3%, *p<0.05). rhVEGF pre‐treatment for 18 hours significantly attenuated apoptosis (8.9%, *p<0.05), however, MM07 displayed no rescue under these conditions.

Supporting info item
