# Supplementary material for: A novel cyclic biased agonist of the apelin receptor, MM07, is disease modifying in the rat monocrotaline model of pulmonary arterial hypertension
Source: Br J Pharmacol. 2019 Apr 1;176(9):1206–21. doi: 10.1111/bph.14603 (PMC6468262; doi:10.1111/bph.14603)
Supplement: Supplementary file 1 — Figure S1: Annexin+/PI‐ stained cells in each condition for each experimental replicate. Staining was consistent throughout experiments as indicated by the limited crossover between the lines, however, basal staining varied between experiments. A matched ANOVA was used so that data trends could be assessed. Figure S2: Rescue of apoptosis of human pulmonary arterial endothelial cells induced by serum and growth factor starvation. Serum and growth factor starvation significantly induced apoptosis relative to the EGM‐2 10%FBS control (9.3%, *p<0.05). rhVEGF pre‐treatment for 18 hours significantly attenuated apoptosis (8.9%, *p<0.05), however, MM07 displayed no rescue under these conditions. [file BPH-176-1206-s001.pdf]

Supplementary figures

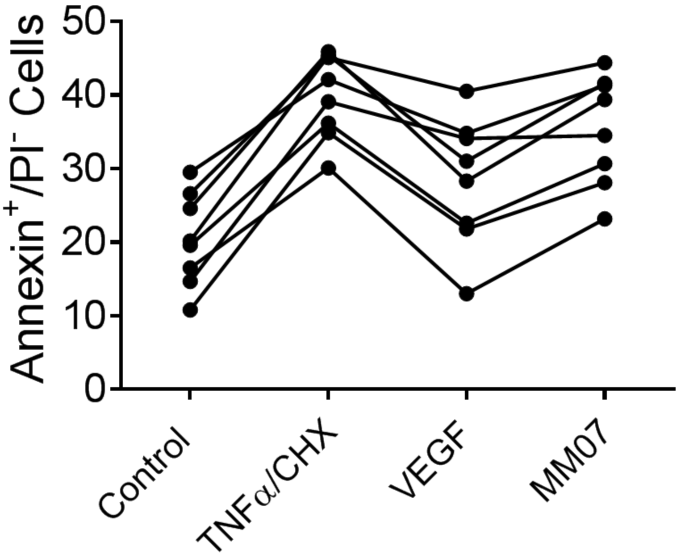

Supplementary figure 1: Annexin^{+}/PI^{-} stained cells in each condition for each experimental replicate. Staining was consistent throughout experiments as indicated by the limited crossover between the lines, however, basal staining varied between experiments. A matched ANOVA was used so that data trends could be assessed.

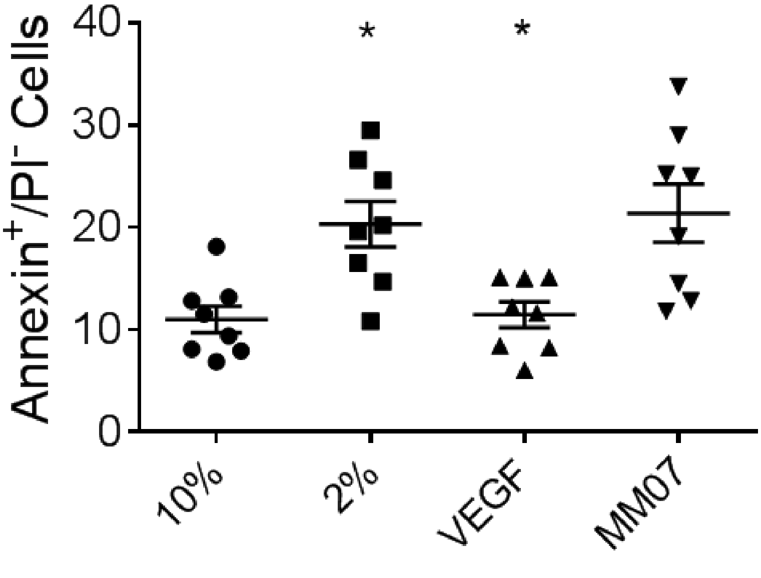

Supplementary figure 2: Rescue of apoptosis of human pulmonary arterial endothelial cells induced by serum and growth factor starvation. Serum and growth factor starvation significantly induced apoptosis relative to the EGM-2 10%FBS control (9.3%, $^{*}p<0.05$). rhVEGF pre-treatment for 18 hours significantly attenuated apoptosis (8.9%, $*p<0.05$), however, MM07 displayed no rescue under these conditions.
