# Supplementary material for: A novel cyclic biased agonist of the apelin receptor, MM07, is disease modifying in the rat monocrotaline model of pulmonary arterial hypertension
Source: Br J Pharmacol. 2019 Apr 1;176(9):1206–21. doi: 10.1111/bph.14603 (PMC6468262; doi:10.1111/bph.14603)
Supplement: Supplementary file 2 — Supporting info item [file BPH-176-1206-s002.zip › Supplementary Videos.docx]

**Supplementary Videos 1-8**

Magnetic resonance imaging (MRI) was carried out to assess cardiac performance on day 20 in a randomly selected sub-set of animals. See the manuscript for experimental methods. Typical examples of cardiac performance following either saline or MCT s.c. exposure on day 0 and subsequent daily i.p. injections of either saline or MM07 (days 1-19) are shown in the Supplementary Videos.

**Video 1.** **Saline Control. Long Axis View.** Cardiac function recorded following saline injection injection s.c. day 0 and daily saline injections i.p day 1-19.

**Video 2.** **Saline Control. Short Axis View.** Cardiac function recorded following saline injection s.c. day 0 and daily saline injections i.p day 1-19.

**Video 3. MM07 Control. Long Axis View.** Cardiac function recorded following saline injection s.c. day 0 and daily MM07 injections i.p day 1-19.

**Video 4. MM07 Control. Short Axis View.** Cardiac function recorded following saline injection s.c. day 0 and daily MM07 injections i.p day 1-19.

**Video 5. MCT Saline. Long Axis View.** Cardiac function recorded following MCT injection s.c. day 0 and daily saline injections i.p day 1-19. Note the enlarged right ventricle, distortion in the interventricular septal wall and less synchronous filling of both ventricles compared to the saline control video.

**Video 6. MCT Saline. Short Axis View.** Cardiac function recorded following MCT injection s.c. day 0 and daily saline injections i.p day 1-19. Note the enlarged right ventricle, distortion in the interventricular septal wall and less synchronous filling of both ventricles compared to the saline control video.

**Video 7. MCT MM07. Long Axis View.** Cardiac function recorded following MCT injection s.c. day 0 and daily MM07 injections i.p day 1-19. MM07 treatment following MCT exposure resulted in cardiac function intermediate between MCT alone and saline control.

**Video 8 MCT MM07. Short Axis View.** Cardiac function recorded following MCT injection s.c. day 0 and daily MM07 injections i.p day 1-19. MM07 treatment following MCT exposure resulted in cardiac function intermediate between MCT alone and saline control.
